# Possible association of rotavirus IgG with cytokine expression levels and dyslipidemia in rotavirus-infected type 1 diabetic children

**DOI:** 10.1007/s11033-022-07573-0

**Published:** 2022-06-22

**Authors:** Rehab G. Khalil, Adel Abdel-Moneim, Amany A. Arafa, Gamal Allam, Waled M. El-Senousy, Doaa Mabrouk

**Affiliations:** 1 Immunology Division, Faculty of Science, Beni-Suef University, Beni-Suef, Egypt; 2 Molecular Physiology Division, Faculty of Science, Beni-Suef University, Egypt. Salah Salem St, 62511 Beni-Suef, Egypt; 3 Department of Water Pollution Research, Environmental Research Division, National Research Centre (NRC), Dokki, Cairo Egypt; 4 Department of Microbiology, Faculty of Medicine, Beni-Suef University, Beni-Suef, Egypt

**Keywords:** Type 1 diabetes, Rotavirus, Anti-rotavirus IgG, Cytokines, Lipid profile

## Abstract

**Background:**

Rotavirus (RV) has been postulated as a viral trigger for the onset of autoimmune disorders, such as type 1 diabetes (T1D). This study aimed to examine the conceivable association of RV IgG with cytokine levels and dyslipidemia in the pathogenesis of pediatric T1D.

**Methods:**

This study included 30 healthy controls and 80 children with T1D who were divided into two groups based on the time since their T1D diagnosis: newly diagnosed (ND ≤ 1 year; n = 30) and previously diagnosed (PD > 1 year; n = 50). ND and PD patients were also separated into negative and positive according to IgG detection (RV IgG^−^, ND^−^, and PD^−^; RV IgG^+^, ND^+^, and PD^+^).

**Results:**

Positive polymerase chain reaction for RVs was evidenced in 7.5% of children with T1D. Anti-RV IgG was 30% and 36% in ND and PD, respectively, compared to healthy controls (2 of 30, 6.6%; *P* < 0.05). Fasting blood sugar and hemoglobin A1c significantly increased in PD^+^ compared to PD^−^. Interferon-γ and interleukin (IL)-15 levels significantly increased. IL-12 and IL-22 mRNA expression was upregulated in ND^+^ patients compared to that in ND^−^ patients. IL-37 mRNA expression was significantly downregulated in ND^−^ and ND^+^ patients compared to that in healthy controls. Total cholesterol and high- and low-density lipoprotein-cholesterol levels were significantly lower in PD^+^ than in PD^−^; whereas triglyceride levels were higher than those in healthy controls.

**Conclusions:**

This study suggested that anti-RV IgG may have a role in the pathogenesis, development, and progression of T1D, and RV infections are implicated in dyslipidemia and inflammation status.

**Supplementary information:**

The online version contains supplementary material available at 10.1007/s11033-022-07573-0.

## Introduction

Type 1 diabetes (T1D) is an autoimmune disease that lasts forever, representing 5–10% of the global diabetes burden [1]. The increasing incidence of T1D cannot be attributed solely to genetics. Environmental triggers and drivers could also play a role. Viruses are a strong contender for environmental causes of T1D, where β-cell damage occurs as a result of the direct lytic effects of viral invasion, resulting in widespread β-cell destruction and absolute insulin insufficiency [2].

Human rotaviruses (RVs) are significant enteric pathogens that infect the epithelial compartment of the small intestine, causing gastroenteritis in infants and children [3]. In 2016, more than 258 million cases of RV infections in children aged < 5 years were recorded worldwide, with an incidence of 0.42 cases per child-year [4]. RV has also been suggested as a viral trigger for the onset of autoimmune illnesses, including celiac disease and T1D [5].

T1D incidence decreased by 3–14% in RV-vaccinated children aged 0 to 4 years in the United States and Australia, supporting RVs as a possible causal agent in the development of T1D [6]. Anti-GAD65 antibody levels in children had a positive connection with anti-RV IgG levels, supporting this theory [7] and epidemiological evidence from a cohort of children that demonstrated a link between an increased risk of T1D and a higher number of gastrointestinal infections, such as RVs [8]. The pathogenic effects of RV on the pancreas *in vivo* were demonstrated in mice, provoking acute pancreatic involution and hyperglycemia [9].

In children, RV infections have been shown to cause RV-specific T-cell and cytokine responses [10]. Interferon (IFN)-γ is a crucial cytokine in various immune responses [11] and has long been considered a contributor to autoimmune T1D. IL-15 is a cytokine that belongs to the IL-2 family and can accelerate T1D *in vivo* [12]. Antigen-presenting cells generate IL-12, a proinflammatory cytokine, and it has been implicated in acceleration; the number of islet-infiltrating Th1 cells increases as diabetes progresses [13]. Although IL-22 is usually regarded as a Th17 cytokine, several studies have suggested/proven that it has an antiviral effect. IL-37 is a newly discovered member of the IL-1 family that acts as a natural suppressor of innate inflammation. A growing body of evidence indicates that IL-37 is involved in a range of inflammatory disorders [14].

Lipids and lipid mediators are increasingly becoming recognized as critical components of several metabolic pathways and cellular processes, particularly in immunology and inflammation [15]. To the authors’ knowledge, no studies have explored the connection between serum lipid profile and RV exposure, and none have looked at it in younger patients. This is essential because atherosclerosis is thought to begin as fatty streaks in the artery wall that emerge throughout childhood [16].

Although enteroviruses (EVs) have been studied in most detail, there is also a small body of research on the potential effects of RV infections on the development of T1D [7]. Furthermore, epidemiological studies alone cannot link RV infections and T1D. Anti-RV IgG in diabetic children’s sera before developing islet autoimmunity or T1D is an incentive to understand disease mechanisms and directly demonstrate the presence of anti-RV IgG in diabetic children’s sera before the onset of islet autoimmunity or T1D. This study aimed to determine whether there is a link between RV IgG antibodies and the development of T1D and to investigate if the cytokine levels and lipid profile of diabetic children have been implicated in RV infections.

## Patients and methods

### Study population

Eighty children with T1D and 30 healthy controls were included in the study. These children sought consultations at Beni-Suef General Hospital (Bani-Suef, Egypt) between October 2017 and January 2018. Healthy controls ranged in age from 3 to 14 years. Children with T1D were divided into two groups depending on how long they have had diabetes: children with newly diagnosed (ND) T1D had been diagnosed with T1D for < 1 year and previously diagnosed (PD) T1D children had been diagnosed for > 1 year. Healthy controls and children with T1D were matched by sex and age. Furthermore, there were 30 children with ND T1D aged 2 to 15 years and 50 children with PD T1D aged 4 to 19 years. None of the participants (both diabetic and nondiabetic) were overweight or obese. In addition, infectious diseases, neoplasms, inflammatory and autoimmune disorders, and allergies were ruled out. Informed written consent was received from the parents of all children, and the study was conducted in compliance with the Declaration of Helsinki, and the Hospital Committee provided its approval (BNS/2019/8).

### Amplification *in vitro*

The total RNA of RVs was extracted from serum using the BIOZOL total RNA extraction reagent (BIOFLUX, Japan) from 140 µl of the sample, according to the manufacturer’s instructions. RV detection using nested polymerase chain reaction (PCR) employed a VP6-coding gene fragment in RVs (Group A). According to Iturriza-Gomara et al. [17], the forward VP6-F primer 5′-GACGGNGCNACTACATGGT-3′ and reverse primer VP6-R 5′-GTCCAATTCATNCCTGGTGG-3′ were used for RV PCR. According to Gallimore et al. [18], nested PCR amplification of the target RV VP6 fragment was conducted using the forward primer VP6-NF 5′-GCTAGAAATTTTGATACA-3′ and reverse primer VP6-NR 5′-TCTGCAGTTTGTGAATC-3′. Electrophoresis of PCR products (10 µl) on 3% agarose gels was performed (Panreac-Spain). At 42 °C for 45 min, cDNA was produced using 50 U expand RT (Roche, Indianapolis, IN, USA). Initial denaturation at 94 °C for 5 min was followed by 30 cycles of 94 °C for 1 min, 50 °C for 2 min, and 72 °C for 2 min. The second round of multiplex PCR was carried out as reported previously [19].

### Anti-RV IgG determination

The classic enzyme-linked immunosorbent assay (ELISA) RV IgG kit was purchased from Serion GmbH (Würzburg, Germany). The procedure was carried out according to the manufacturer’s instructions. IgG levels were allocated to RV patients as positive (above the cutoff value; anti-RV IgG^+^) or negative (below the cutoff value; anti-RV IgG^−^). The cutoff value was derived using the control’s mean [2⋅ + standard deviation (SD)]. IgG was also utilized to divide children with ND and PD T1D into negative and positive groups (anti-RV IgG^−^; ND^−^ and PD^−^; anti-RV IgG^+^; ND^+^ and PD^+^).

### Biochemical assays

Fasting blood sugar (FBS) and hemogloblin A1c (HbA1c) assays were performed on participants. SPINREACT (Girona, Spain) and MyBiosource (California, USA) commercial kits were used. The C-peptide ELISA kit was provided by DRG Diagnostics (Marburg, Germany). Total cholesterol (TC), triglycerides (TGs), and high-density lipoprotein-cholesterol (HDL-C) levels were measured using a colorimetric enzymatic method (Spain). The Friendewald et al. [20] formula was used to estimate the low-density lipoprotein-cholesterol (LDL-C) levels. Sandwich ELISA kits (R&D Systems, Minneapolis, MN, USA) were used to detect IFN-γ and IL-15 levels in the blood. With each kit, all steps were completed according to the manufacturer’s recommendations.

### Isolation of RNA with real-time PCR for IL-12, IL-22, and IL-37 mRNA expression

Total RNA was extracted from blood samples of all groups using TRIzol reagent (MBI Fermentas, Germany), and cDNA was synthesized using the High-Capacity cDNA Reverse Transcription Kit (Invitrogen, Germany) according to the manufacturer’s instructions. In a 20 µL system, real-time PCR utilized 10 µL of 1⋅ SsoFast EvaGreen Supermix (Bio-Rad, Hercules, CA, USA), 2 µL cDNA, 6 µL RNase/DNase-free water, and 500 nM of the following PCR primer pair sequences:IL-12 forward 5′-TCAAAAGGAGGCGAGGTTC–3 ′ and reverse 5 ′ A T C AGAACCTAACTGCAGGG-3′, IL-22 forward 5′-AAAATGAGTCCGTG ACCAAAATGC-3′ and reverse 5′-ACACAATTGTTTTGTCTTAGTAGAGTTCAGAT-3′, and IL-37 forward 5′-CAGCCTCTGCGGAGAAAGGAAGT-3′and reverse 5′GTTCTCCTTCTTCAGCTGAAGGGA TGGAT-3′. The thermal cycler conditions were as follows: 30 s at 95 °C, followed by 40 cycles of 5 s at 95 °C and 10 s at 60 °C. For each reaction, a 65 to 95 °C ramp was performed, followed by a melting curve analysis. The relative amount of mRNA was measured, and the threshold length at which the fluorescent signal exceeded an arbitrarily selected threshold close to the midpoint of the log-linear amplification step was estimated for each operation. Data from amplification were examined using the manufacturer’s software and Livak and Schmittgen methods [21]. The variables were standardized to β-actin.

### Statistical analysis

The means and SDs were used to express the findings. The Statistical Package for Social Sciences version 22 software system (Chicago, IL, USA) for Windows was used to analyze data. A one-way analysis of variance and a least significant difference t-test were employed to compare the groups and detect significant differences. Pearson’s correlations were utilized to determine the links between the variables. *P* < 0.05 was used as the significance level.

## Results

### Demographic information

Gender, age, and family history values did not differ significantly (*P* > 0.05) between ND and PD T1D compared to healthy controls (Table 1). Although FBS and HbA1c (%) levels in children with T1D (in both groups) were significantly higher (*P* < 0.001) than those in healthy controls, there was no difference in HbA1c between children with ND T1D and those with PD T1D (*P* > 0.05). In contrast, C-peptide concentrations were significantly (*P* < 0.001) lower in both cases than in healthy controls. Furthermore, C-peptide levels were significantly lower (*P* < 0.001) in children with PD T1D than in children with ND T1D (Table 1). Body mass index (BMI) showed no significant difference (*P* > 0.05) between children with PD T1D and healthy controls, but it did show an increase in children with ND T1D versus healthy controls (Table 1).

**Table 1:** Demographic data of healthy controls, newly diagnosed T1D, and previously-diagnosed T1D

	Healthy controls (n = 30)	Newly-diagnosed (ND) T1D(n = 30)	Previously-diagnosed (PD) T1D (n = 50)	P value
Gender, no. (%) Male Female	12(40) 18(60) ^a^	14(46.6) 16(53.4) ^a^	20(40) 30(60) ^a^	p > 0.05
Age (Year)	(8.4 ± 2.84)^a^	(9.1 ± 4.4)^a^	(10.78 ± 3.5)^b^	p < 0.05
Family history, no. (%)	1(3.4) ^a^	3(10) ^ab^	9(18) ^b^	p < 0.05
RNA of RVs	1(3.3%) ^a^	5(16.7%) ^b^	2(4%) ^a^	p < 0.05
Anti-Rota-IgG^+^ no (%)	2(6.6%) ^a^	9(30%) ^b^	18(36%) ^b^	p < 0.05
Duration of diabetes (months)	------------	(7.72 ± 4.423) ^a^	(51.4 ± 20.749) ^b^	P < 0.001
BMI (Kg/m^2^)	(17.54 ± 2.818)^a^	(19.75 ± 4.480) ^ab^	(19.21 ± 4.45) ^ab^	p > 0.05
FBS (mg/dl)	(95.47 ± 9.25) ^a^	(244.36 ± 46.06)^b^	(200.6 ± 27.99)^c^	P < 0.001
HbA1C (%)	(4.15 ± 0.143) ^a^	(9.27 ± 2.127)^b^	(8.76 ± 1.61)^b^	P < 0.001
C-peptide (ng/ml)	(4.25 ± 0.879) ^a^	(0.621 ± 0.234)^b^	(0.298 ± 0.151)^c^	P < 0.001

Data are expressed as mean ± SD. Means which share the same superscript symbol(s) are not significantly different P > 0.05. FBS: Fasting Blood Sugar HbA1c: glycated hemoglobin BMI: Body Mass Index

### Detection of total RNA of RVs

Positive RV PCR was evidenced in 7.5% of children with T1D (7 of 80) and had nonsignificance (*P* > 0.05) compared to that in healthy controls (2 of 30, 6.6%; Table 1). Between ND and PD T1D groups, positive RV PCR was more frequently detected in children with ND T1D (5 of 30, 16.6%) than in children with PD T1D (2 of 50, 4%; *P* < 0.05; Table 1).

### Detection of anti-RV IgG

The seroprevalence of anti-RV IgG revealed the presence of specific anti-RV IgG in 6.6% (2 of 30) of healthy controls compared to 33.75% (27 of 80) in diabetic children (*P* < 0.05; Table 1). Anti-RV IgG was 30% (9 of 30) positive in ND children, but PD T1D showed 36% (18 of 50) compared to that in healthy controls (*P* < 0.05; Table 1).

### Serum levels of FBS, HbA1c, and C-peptide

**Fig. 1:**
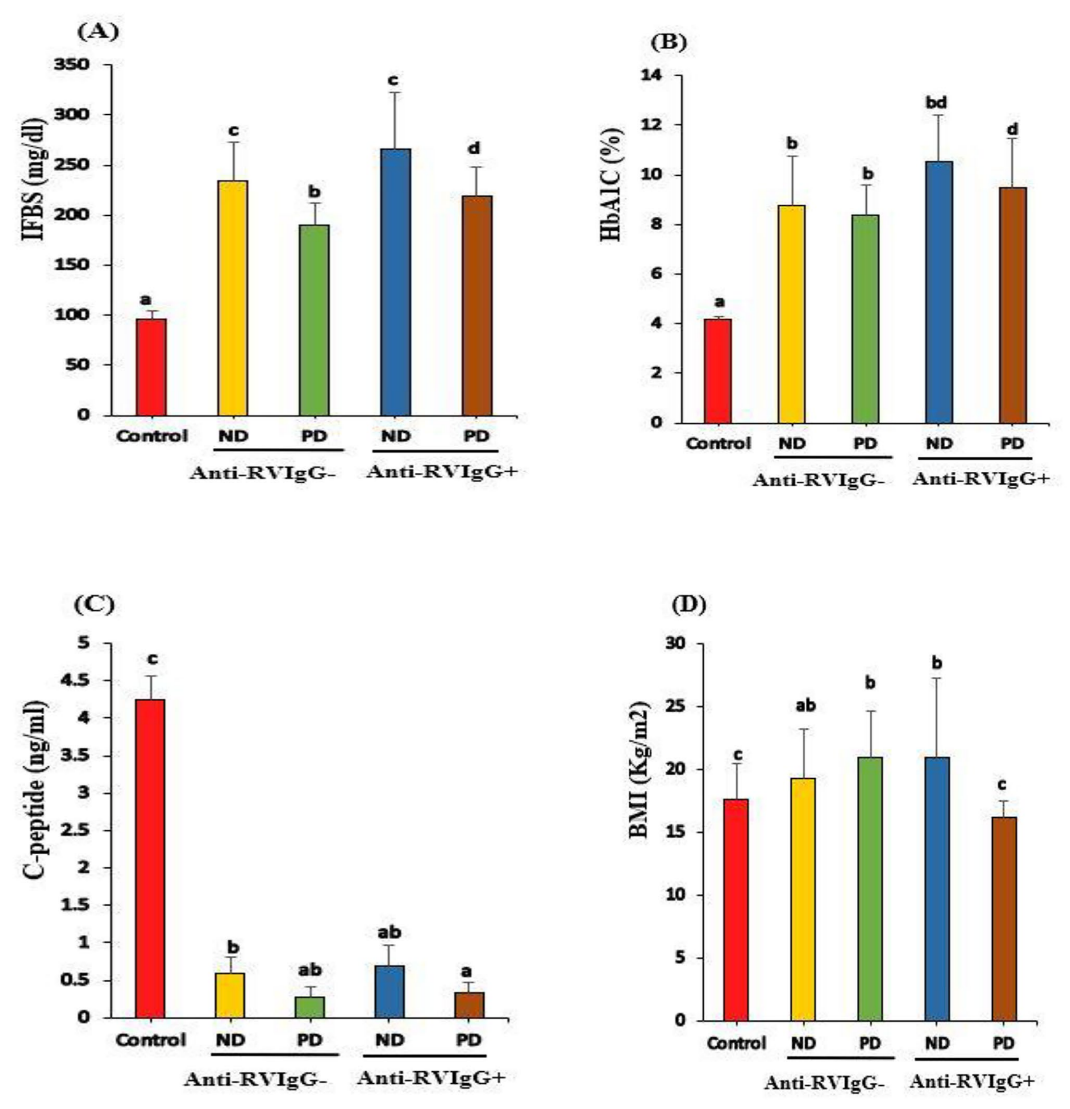
FBS(A), HbA1C(B) and C-peptide levels of healthy controls, Anti-RV IgG^−^(ND^−^ and PD^−^) and children with Anti-RV IgG^+^ (ND^+^ and PD^+^) T1D children. Means which share the same superscript symbol(s) are not significantly different P > 0.05 FBS: Fasting Blood Sugar HbA1c: glycated hemoglobin ND: newly diagnosed PD: previously diagnosed

FBS and HbA1c (%) levels were elevated markedly in children with T1D in both groups (anti-RV IgG^−^ and IgG^+^) compared to those in healthy controls (*P* < 0.001). In contrast, C-peptide concentrations were significantly (*P* < 0.001) lower, including both T1D groups. FBS levels were not statistically different between ND^−^ and ND^+^ (*P* > 0.05), whereas PD^+^ levels were significantly higher than PD^−^ (*P* < 0.05; Fig. 1 A). HbA1c (%) levels were significantly increased in PD^+^ compared to those in PD^−^ (*P* < 0.05; Fig. 1B). C-peptide levels and BMI showed a significance decrease (*P* < 0.05) in PD^+^ compared to those in PD^−^ (Fig. 1C and D). Particularly, FBS and HbA1c (%) levels were elevated markedly (*P* < 0.001) in children with positive RV PCR compared to those in healthy controls and negative RV PCR, however, C-peptide concentrations were lowered significantly (*P* < 0.001) (Table S).

### Cytokine levels and mRNA expression

**Fig. 2:**
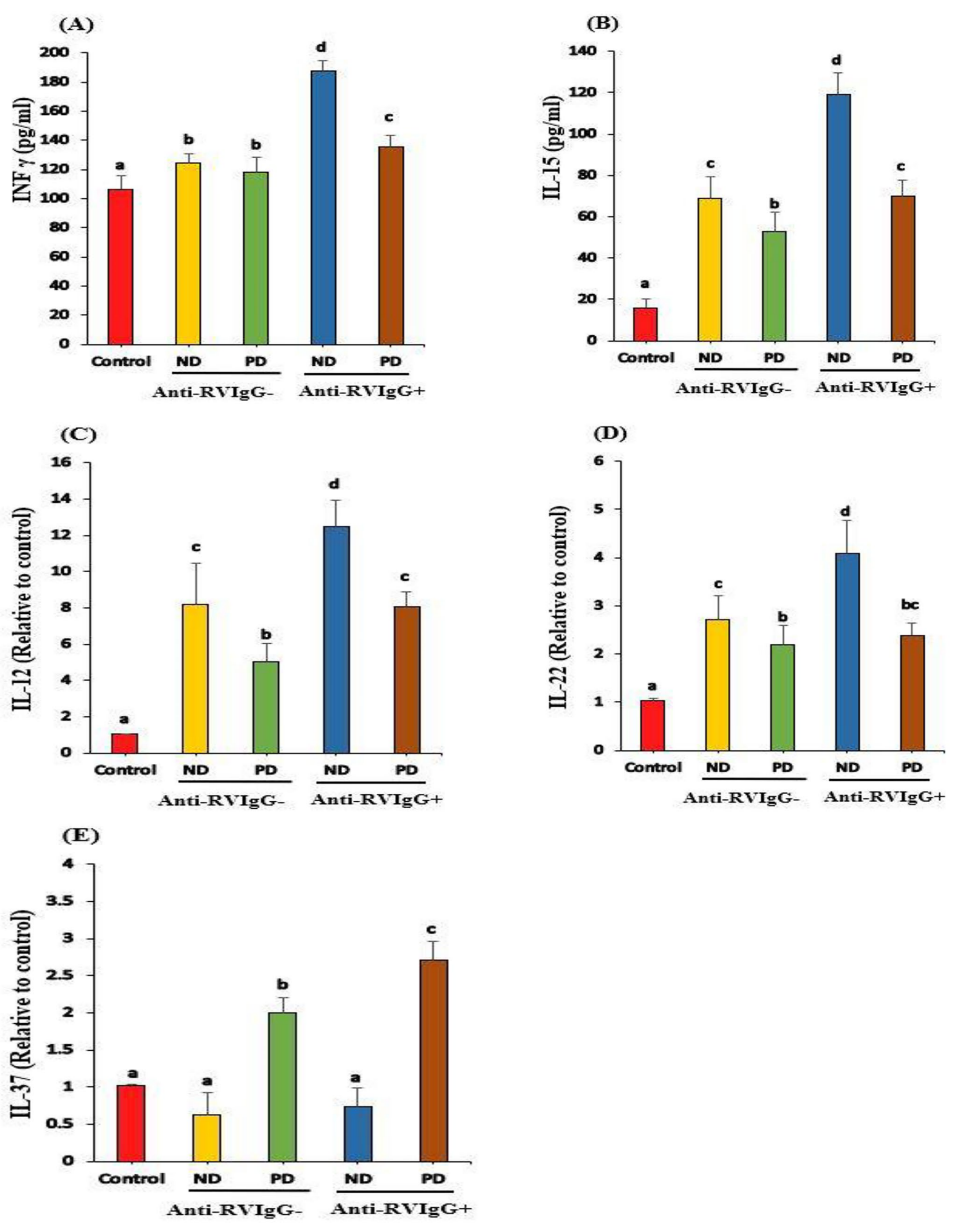
IFN- γ (A), IL-15(B) PCR IL-12 (A), PCR IL-22 (B), and PCR IL-37 (C) levels of healthy controls, Anti-RV IgG^−^(ND^−^ and PD^−^) and children with Anti-RV IgG^+^ (ND^+^ and PD^+^) T1D children. Means which share the same superscript symbol(s) are not significantly different P > 0.05

IFN-γ and IL-15 levels were significantly increased in both T1D groups (anti-RV IgG^−^ and IgG^+^) compared to those in healthy controls (Fig. 2A and B). However, these levels in ND^+^ were significantly (*P* < 0.05) increased compared to those in ND^−^ (Fig. 2A and B). Compared to those in healthy controls, IL-12 production and IL-22 mRNA expression were considerably (*P* < 0.05) increased in all T1D children (Fig. 2C and D). IL-12 mRNA expression levels were clearly (*P* < 0.05) elevated in ND^+^ compared to those in ND^−^, whereas IL-22 mRNA expression showed a nonsignificant change between PD^+^ and PD^−^ (Fig. 2C and D). IL-37 mRNA expression was significantly (*P* < 0.05) downregulated in ND^−^ and ND^+^ T1D children compared to that in healthy controls (*P* < 0.05) but upregulated in PD^−^ and PD^+^ children compared to that in healthy controls (*P* < 0.05; Fig. 2E).

### Serum levels of lipid profile

**Fig. 3:**
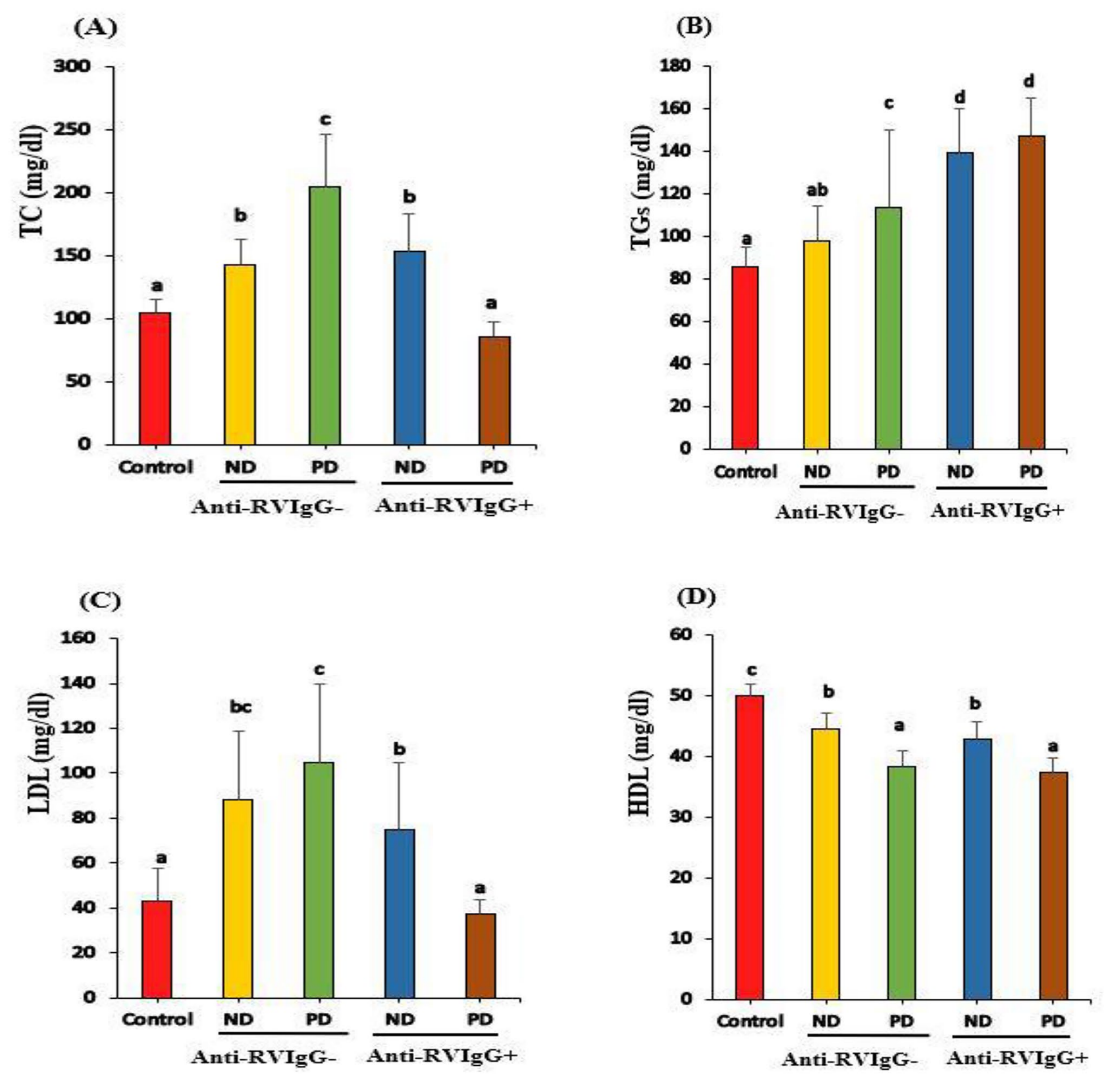
T.C(A), T.G(B), LDL(C) and HDL(D) levels of healthy controls, Anti-RV IgG^−^(ND^−^ and PD^−^) and children with Anti-RV IgG^+^ (ND^+^ and PD^+^) T1D children. Means which share the same superscript symbol(s) are not significantly different P > 0.05 T.C: Total cholesterol, T.G: Triglycerides, LDL: Low-Density Lipoprotein and HDL: High-Density Lipoprotein. ND: newly diagnosed PD: previously diagnosed

TC and LDL levels showed a significant increase in ND^−^, PD^−^, and ND^+^ compared to those in healthy controls (*P* < 0.05; Fig. 3A and C) but a high decrease in PD^+^ compared to PD^−^ (*P* < 0.05; Fig. 3 A and C), However, TG levels have markedly (*P* < 0.05) increased in both ND^+^ and PD^+^ compared to those in ND^−^ and PD^−^ and healthy controls (*P* < 0.05; Fig. 3B). In contrast, HDL levels decreased in both diabetic groups (anti-RV IgG^−^ and IgG^+^) compared to those in healthy controls (*P* < 0.05). However, there was no statistically significant difference between ND^−^ and ND^+^ and between PD^−^ and PD^+^ (*P* > 0.05; Fig. 3D).

### Anti-RV IgG correlations

**Fig. 4:**
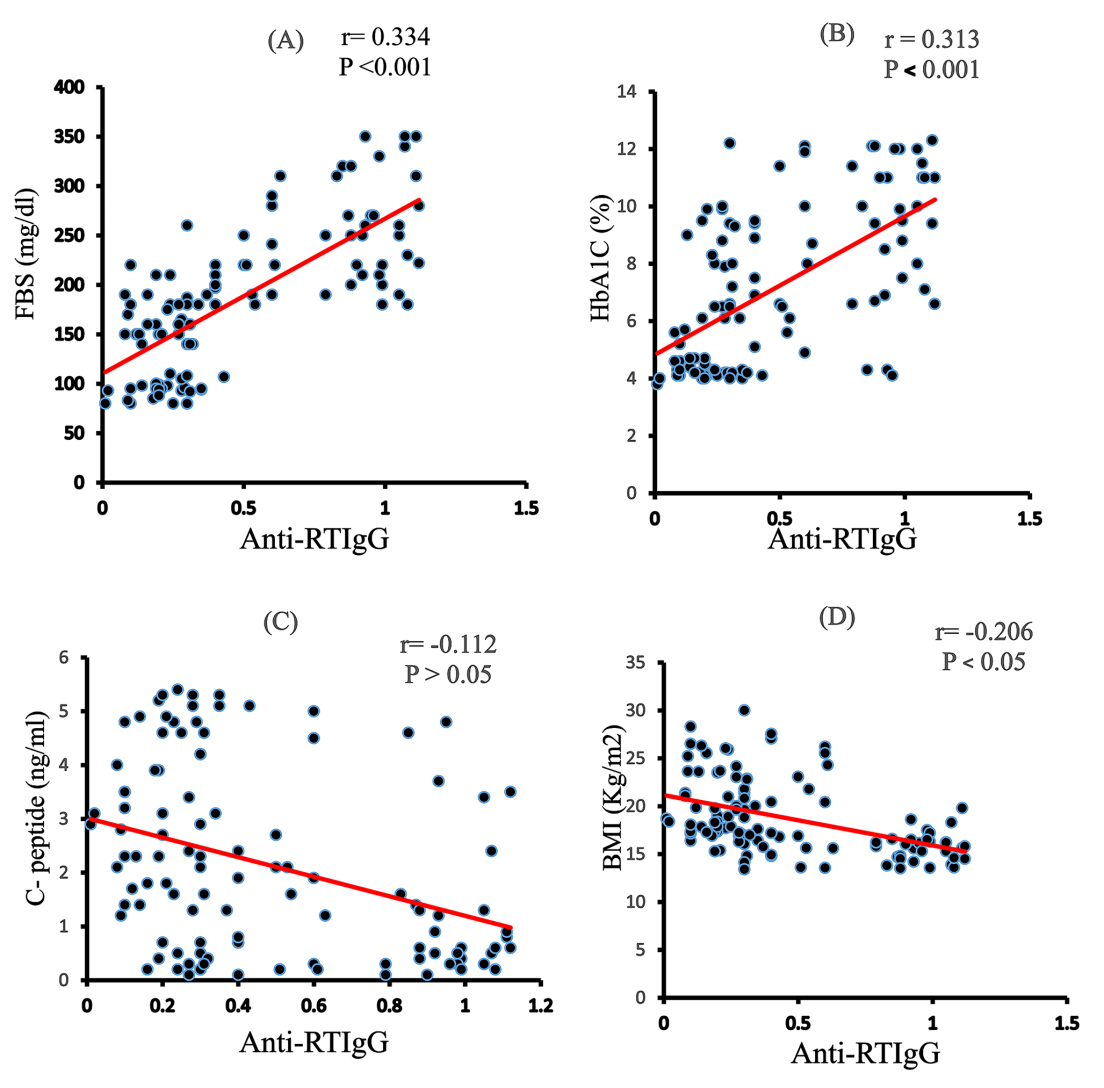
Correlations between the levels Anti-RV IgG and (A)FBS, HbA1c (b), and C-peptide (C), Values were considered significantly different at*P < 0.05, **P < 0.01and***P < 0.001

**Fig. 5:**
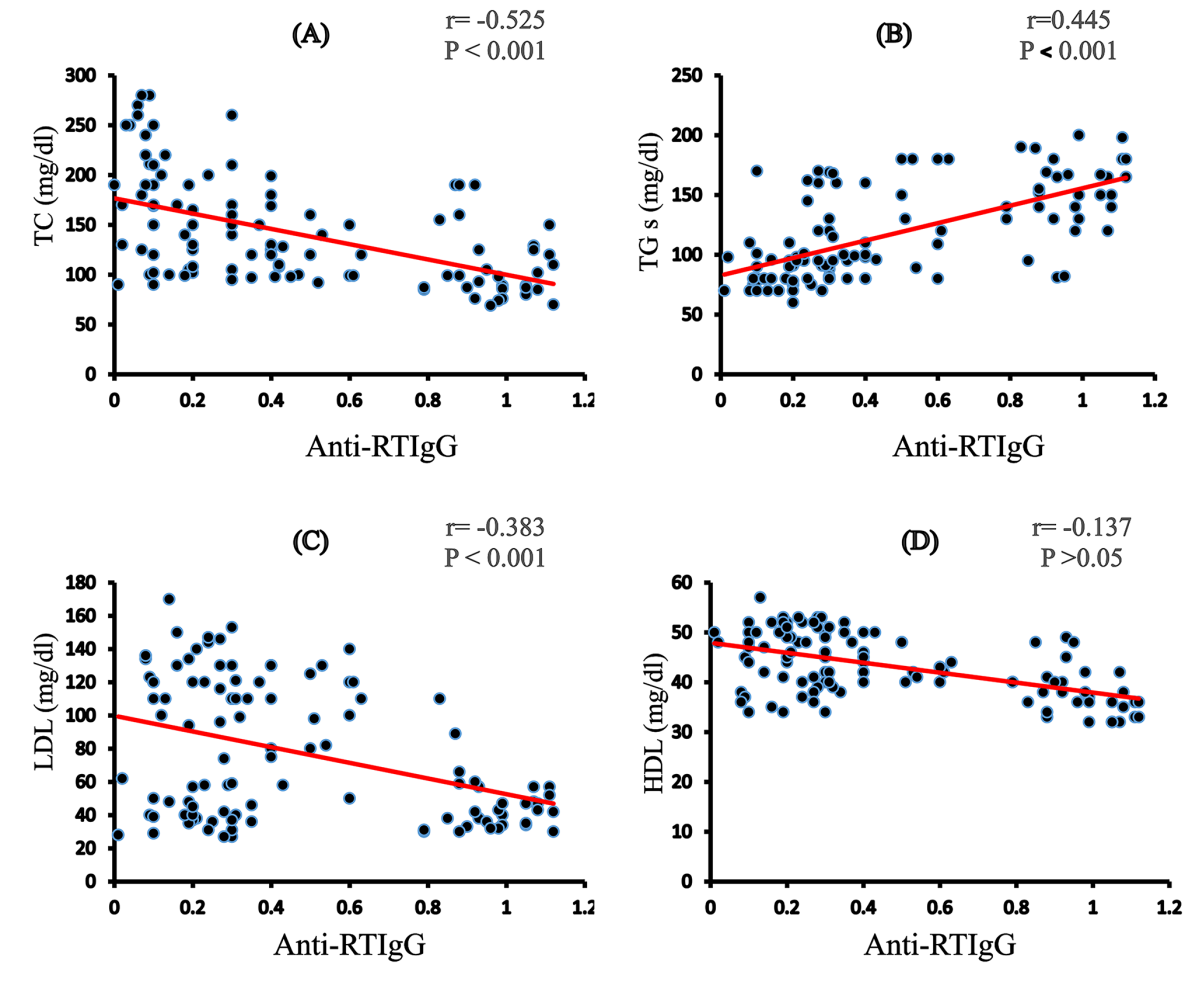
Correlations between the levels Anti-RV IgG and (A)T.C, T.G (B), LDL (C), and HDL (D), Values were considered significantly different at*P < 0.05, **P < 0.01and***P < 0.001

**Fig. 6:**
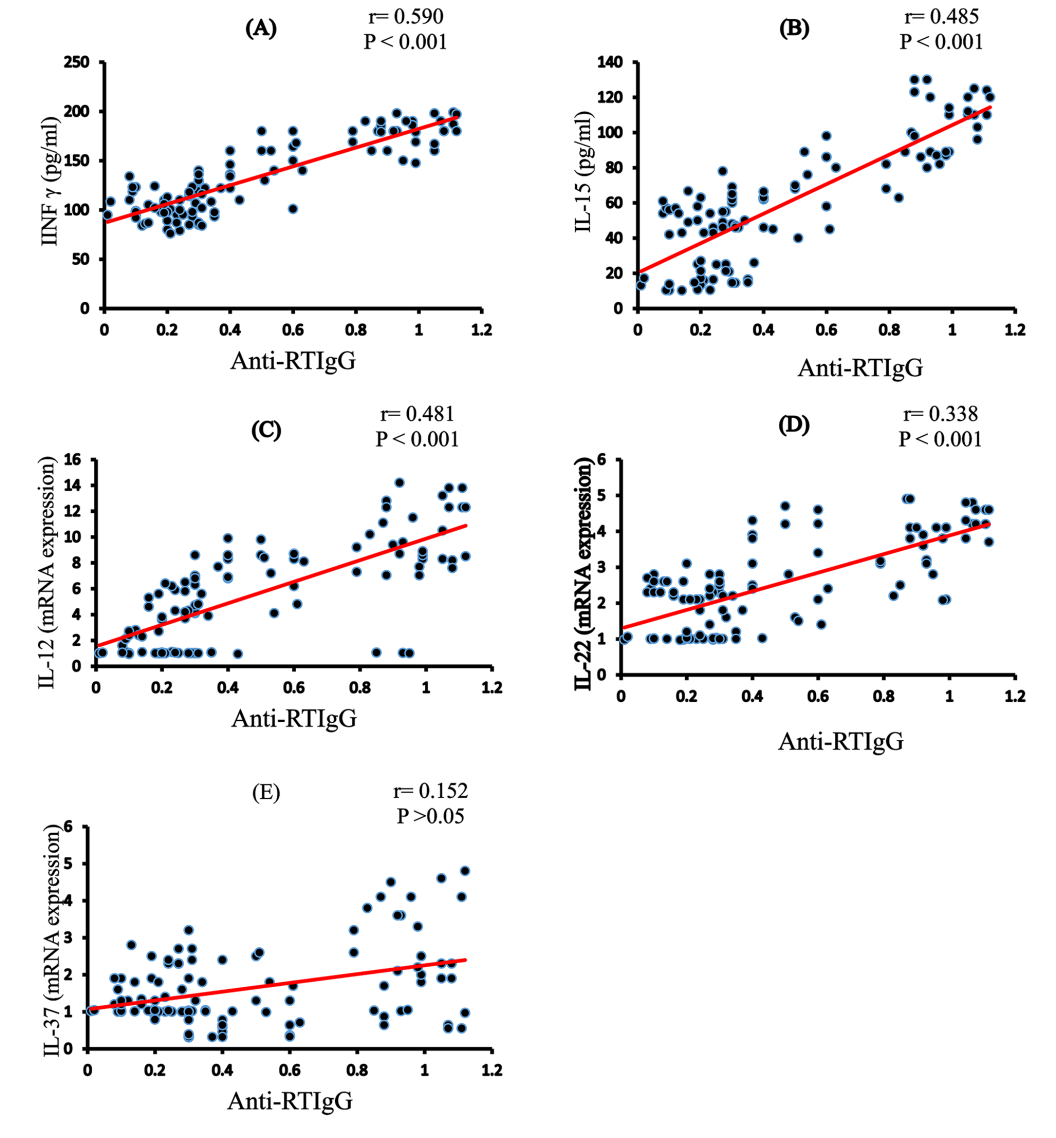
Correlations between the levels Anti-RV IgG and IFN- γ (A), IL-15(B) PCR IL-12 (C), PCR IL-22 (D), and PCR IL-37 (E) Values were considered significantly different at*P < 0.05, **P < 0.01and***P < 0.001

In diabetic children, anti-RV IgG was positively correlated with FBS (*r* = 0.344, *P* < 0.001), HbA1c (*r* = 0.313, *P* < 0.001), and TGs (*r* = 0.445; *P* < 0.001) but negatively correlated with C-peptides (*r* = − 0.112, *P* < 0.05), BMI (*r* = − 0.206, *P* < 0.05), TC (*r* = -0.525, *P* < 0.001), LDL (*r* = − 0.383, *P* < 0.001), and HDL (*r* = − 0.137, *P* < 0.05; Figs. 4 and 5). Anti-RV IgG was positively correlated with all measured cytokines, IFN-γ levels (*r* = 0.590, *P* < 0.001), IL-15 levels (*r* = 0.485, *P* < 0.001), IL-12 mRNA expression (*r* = 0.481, *P* < 0.001), mRNA expression of IL-22 (*r* = 0.338, *P* < 0.001), and IL-37 mRNA expression (*r* = − 0.152, *P* < 0.05; Fig. 6).

## Discussion

RV was chosen to estimate the prevalence of RV infections and the seroprevalence of RV IgG antibodies among diabetic children to find the relationship between infectivity and T1D. The incidence of RV infections in T1D children based on RV RNA detection in serum was 7.5% of positive cases in this study. Moreover, our study revealed that FBS and HbA1c (%) levels were elevated markedly in children with positive RV PCR compared to those of negative RV PCR, however, C-peptide levels were lowered significantly. The possible role of RV in the etiology of T1D has been reported in several studies, RV infection triggers pancreatic apoptosis in mice, and RV peptides display molecular mimicry with T-cell epitopes in pancreatic β-cell auto-antigens. Also, maternal RV infections during pregnancy may damage the fetal islet cells and trigger the cascade of events leading to TID [22]. To reduce the risk of infection, vaccines are strongly recommended. However, there are few studies regarding the immunogenicity and efficacy of vaccines against T1D [23]. RV vaccination may be able to attenuate the ever-increasing incidence of childhood T1D, with the exception of certain subpopulations with a different degree of susceptibility[24]. The chance of an infection by viruses found in the environment could be due to the etiology of T1D, such as EVs [25], cytomegalovirus [26], Epstein–Barr virus [27], parechovirus [28], influenza virus [29], mump virus [30], rubella virus [31], and human endogenous retrovirus [32]. In contrast, the incubation for RV is only about 2 days, and infection can extend up to 10 days. In addition, two RV licensed vaccines in 2006 (Rotarix and RotaTeq) for the pediatric population worldwide have shown a significant impact on the burden of RV infections [33]. However, continuing studies are looking into the impact of these RV vaccines on extraintestinal symptoms and the development of autoimmune disorders [5]. Importantly, another explanation is that various factors, including genetics, immunological regulation, age, and environment, all played a role in the autoimmune response [34].

In the current study, RV IgG antibodies were present in diabetic children with highly specific IgG response rates (30% and 36% in ND and PD T1D, respectively) with statistically significant (*P* < 0.01) variation compared to that in healthy controls. Elevated IgG levels in children could indicate the same virus infection [35]. However, RV is extremely contagious among children, and numerous bouts of gastroenteritis in the first year of life can lead to many infections with different virus strains [6]. In addition, IgG levels among anti-RV IgG^+^ (PD^+^; >1 year of diagnosis) who may have been subjected to recurrent infections were substantially greater than anti-RV IgG^+^ (ND^+^; <1 year of diagnosis). The dependency of IgG strength on acute and recurring infections, the last of which would raise insulin autoantibodies (IAA) and islet cell autoantibodies (ICA), supported the view of increased continuing autoimmune and overt diabetes after viral infections. In addition, IgG and autoantibodies have a positive association [7]. Detection of antiviral antibodies (IgM or IgG) refers to a viral infection, and inflammation occurs. This inflammation results in the activation of autoreactive T cells and, as a consequence, an autoimmune response [36]. Also, antibodies against various viruses can increase the infection of target cells [37]. This phenomenon is called antibody-dependent enhancement of infection. Overall, these findings supported the idea of anti-RV IgG infection that delivers information like a virus trigger for islet autoimmunity regarding the mechanism involved while emphasizing the importance of an individual’s genetic background.

In this study, HbA1c levels have increased in the two diabetic groups (anti-RV IgG^+^ and IgG^−^). However, diabetic anti-RV IgG^+^ showed a further increase compared to anti-RV IgG^−^ and PD^+^ compared to ND^+^. Thus, RV IgG is accompanied by the appearance of autoantibodies and, as a result, accelerated β-cell damage and decreased insulin production, as seen by lower C-peptide in the IgG^+^ diabetes group. The efficient involvement of RV in inhibiting insulin secretion has been attributed to the depletion of its granule reserves and hence insulin secretion suppression [38]. These data were confirmed by a significant positive correlation between anti-RV IgG and both FBS and HbA1c levels but a negative correlation with C-peptide. Maintaining repeated viral infections exacerbates chronic autoimmunity and T1D consequences. Recurrent infections may infiltrate more β-cells. As a result, the number of activated CD8^+^ T cells and autoantibody levels increases. This study showed proinflammatory cytokine (IFN-γ, IL-15, IL-12, and IL-22) levels increased in the anti-RV IgG^+^ group compared to those in the anti-RV IgG^−^. These cytokines are associated not only with T1D [13] but also with viral infections [39, 40]. Indeed, in T1D-associated autoimmunity, the orientation of immune response to the Th1 phenotype by IFN-γ production was observed during viral infections. Increased levels of IFN-γ, IL-12, and IL-22 in the anti-RV IgG^+^ group compared to those in the anti-RV IgG^−^ group suggested a predominance of Th1 and Th17 immune response. Both IFN-γ and IL-12 have been implicated in the activation of natural killer (NK) cells, which are responsible for pancreatic apoptosis [41]. Thus, NKs with the help of IFN-γ and IL-12 initiated β-cell injury before activating CD8^+^ T cells and producing antiviral antibodies. In this study, these cytokines were increased in anti-RV IgG^+^ ND^+^ compared to those in PD^+^. IL-15 also promotes the proliferation and activation of NK and CD8^+^ T cells, and IL-15-activated NK cells can suppress Foxp3 expression in CD4^+^ Tregs [12]. The roles of IL-22, a member of the IL-10 family, in the inflammatory microenvironment are mutable; thus, it can yield either protective or pathogenic functions [42]. In pancreatic β-cells, IL-22 increases the expression of antiapoptotic proteins Bcl-2 and Bcl-xL and regenerative proteins Reg1 and Reg2 [43]. In this study, IL-12 mRNA expression was upregulated in ND^+^ compared to that in ND^−^, whereas IL-22 mRNA expression showed a nonsignificant change between PD^+^ and PD^−^ where, in intestinal RV infections, innate lymphocytes are also a key source of IL-22. These data showed that IL-22 has an antiviral effect by controlling RV infections [40].

Human peripheral blood mononuclear cells, macrophages, epithelial cells, and activated B cells can produce IL-37, an anti-inflammatory cytokine [44]. Here, IL-37 decreased in anti-RV IgG (ND^−^ and ND^+^) with nonsignificance compared to control, a finding that agreed with that by reported by Harms et al. [45], who revealed that IL-37 levels showed a nonsignificant difference between control and T1D populations but increased in anti-RV IgG (PD^−^ and PD^+^ compared to control, ND^−^, and ND^+^). This finding could be attributed to low IL-37 levels that reduce the host’s ability to dampen inflammation by inhibiting downstream proinflammatory signal kinases, such as mammalian target of rapamycin and mitogen-activated protein kinase, resulting in increased inflammatory cytokine levels [46]. Many Toll-like receptor (TLR) ligands, such as TLR2 and TLR4, can stimulate IL-37 to be produced in response to infections [47]. Mer and PTEN, two anti-inflammatory signaling molecules, can also be activated [48]. Overall, there was a notable association between antiviral antibodies, Th1 and Th17 cytokines, and inflammation and cell death in RV infections, which was validated by a statistical correlation that revealed increased levels of IFN-γ and IL-15 and upregulation of IL-12 and IL-22 mRNA expression with anti-RV IgG. As a result of RV entrance into the cell, more β-cell antigens were exposed to the immune system response, resulting in autoimmunity [49].

Another interesting finding of this study was an increase in TG levels in both ND^+^ and PD^+^ relative to ND^−^, PD^−^, and healthy controls. Indeed, rather than the initial immune response, high TG levels are linked to the development of secondary immunological reactions and the generation of memory cells [50] showed that inflammation increases angiopoietin-like protein 4, an inhibitor of lipoprotein lipase activity, further blocking the metabolism of TG-rich lipoproteins. Antibodies to lipoprotein lipase have been reported in systemic lupus erythematosus and are associated with increased TG levels [51]. TC and LDL levels showed a significant increase not only in ND^−^ and PD^−^ but also in ND^+^ compared to those in healthy controls but decreased significantly in PD^+^ compared to those in PD^−^; however, HDL levels decreased in all diabetic groups. These results agreed with Munger et al. [52]. The inhibition of fatty acid biosynthesis suppressed viral replication for both human CMV (HCMV) and influenza A virus. In addition, patients with acute EBV infections had lower TC, LDL-C, HDL-C, apoAI, and apoB and higher TC levels than their age- and sex-matched controls [53]. Recently, LDL-C, HDL-C, and TC levels were significantly lower in COVID-19 patients than in healthy controls, although TG levels were higher [54]. Of note, both HCMV and EBV, as cited above, were implicated in T1D. Also, these results were confirmed by a negative correlation between anti-RV IgG and TC, LDL, and HDL levels but correlated positively with TGs. With increasing serum TG levels, there is an increase in the content of virus-specific antibodies.

The limitation of this study consists of the small number of included subjects and the lack of autoantibody detection, such as insulin autoantibody, islet cell antibody, and glutamic acid decarboxylase. Also, this study lacks data on the protein levels of some cytokines. All these limits should be manipulated in future studies.

## Conclusions

The study suggested that anti-RV IgG may have a role in the pathogenesis, development, and progression of T1D and subsequent inflammation and development of T1D in children. Increased IFN-γ, IL-15, IL-12, and IL-22 levels with decreased IL-37 levels in ND^+^ compared to those in PD^+^ were associated with RV-induced T1D. In addition, TC, HDL, and HDL were all lowered during (due to) RV infections in diabetic children for the first time, whereas TG was elevated.

## Electronic supplementary material

Below is the link to the electronic supplementary material.

Supplementary Material 1

## Data Availability

All data generated or analysed during this study are included in this published article.
