# Supplementary material for: Possible association of rotavirus IgG with cytokine expression levels and dyslipidemia in rotavirus-infected type 1 diabetic children
Source: Mol Biol Rep. 2022 Jun 22;49(8):7587–99. doi: 10.1007/s11033-022-07573-0 (PMC9216291; doi:10.1007/s11033-022-07573-0)
Supplement: Supplementary file 1 — Supplementary Material 1 [file 11033_2022_7573_MOESM1_ESM.docx]

**Table S:** Comparison between healthy control, T1D –RV^-^ and T1D – RV^+^ PCR regarding diabetes profile

|  | **Control**  **(n=30)** | **T1D – RV¯**  **(n=73)** | **T1D – RV^+^**  **(n=7)** | **P value** | |
| --- | --- | --- | --- | --- | --- |
| **Gender, no. (%)**  Male  Female | 12(40)  18(60) | 31(42.5)  42(57.5) | 3(42.8)  4(57.2) | P>0.05 | |
| **Age (year)**  Range  Mean±SD  **Duration of diabetes(months)**  Range  Mean±SD | 3-14  (8.4±2.84)^a^  ---------- | 3-15  (9.5±3.1)^a^  1-84  (17.4±17.8)^b^ | 3-16  (9.5±3.3)^a^  1-84  (16.8±18.1)^b^ | P>0.05  P>0.05 | |
| **FBS (mg/dl)**  **Range**  Mean±SD | 80-110  (95.4±9.25)^a^ | 120-200  (168.7±21.3)^b^ | 180-295  (232.2±33.56)^c^ | p< 0.001 |  |
| **HbA1C (%)**  Range  Mean±SD | 3.5-4.4  (4.15 ±0.143) ^a^ | 3.6-8.2  (6.36±1.36) ^b^ | 6.1-14  (10.46±2.2.6) ^c^ | p< 0.001 |  |
| **C-peptide (ng/ml)**  Range  Mean±SD | 2.7-5.6  (4.25±0.879) ^a^ | 0.1-0.8  (0.56±0.219) ^b^ | 0.1-0.4  (0.113±0.135) ^c^ | p< 0.001 |  |

Data are expressed as mean ± SD. Means which share the same superscript symbol(s) are not significantly different P>0.05.
